# N2A: a computational tool for modeling from neurons to algorithms

**DOI:** 10.3389/fncir.2014.00001

**Published:** 2014-01-24

**Authors:** Fredrick Rothganger, Christina E. Warrender, Derek Trumbo, James B. Aimone

**Affiliations:** Cognitive Modeling Department, Sandia National LaboratoriesAlbuquerque, NM, USA

**Keywords:** neuroinformatics, computational modeling, computational neuroscience, structural plasticity, biologically realistic modeling

## Abstract

The exponential increase in available neural data has combined with the exponential growth in computing (“Moore's law”) to create new opportunities to understand neural systems at large scale and high detail. The ability to produce large and sophisticated simulations has introduced unique challenges to neuroscientists. Computational models in neuroscience are increasingly broad efforts, often involving the collaboration of experts in different domains. Furthermore, the size and detail of models have grown to levels for which understanding the implications of variability and assumptions is no longer trivial. Here, we introduce the model design platform *N2A* which aims to facilitate the design and validation of biologically realistic models. N2A uses a hierarchical representation of neural information to enable the integration of models from different users. N2A streamlines computational validation of a model by natively implementing standard tools in sensitivity analysis and uncertainty quantification. The part-relationship representation allows both network-level analysis and dynamical simulations. We will demonstrate how N2A can be used in a range of examples, including a simple Hodgkin-Huxley cable model, basic parameter sensitivity of an 80/20 network, and the expression of the structural plasticity of a growing dendrite and stem cell proliferation and differentiation.

## Introduction

Computational neuroscience methods for constructing and simulating biologically realistic models have increasingly been recognized as important for understanding the function of complex neural circuits. The role for computational tools will continue to grow in the near future, with significant policy efforts such as the EU Human Brain Project (Markram, 2012) and the proposed Brain Activity Map (Alivisatos et al., 2013). These programs emphasize the high-throughput collection of neural data through both connectomics research and large scale physiology measurements of neuronal behavior in circuits. While the role of computational tools for modeling and simulation is increasingly recognized, the path from this raw data to interpretable model results is unclear.

**Figure 1:**
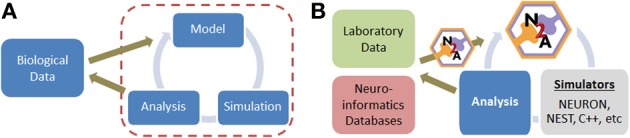
**(A)** Simple overview of computational modeling process and relationship to neurobiological data. **(B)** Illustration of where N2A tool contributes to neural modeling studies; we envision that N2A will eventually be capable of mapping into a wide range of common neural simulation platforms.

Constructing neural simulations typically involves several distinct stages once a conceptual approach has been established (Figure 1A). **(1)** Relevant data from the biological world must be identified, filtered, and represented in a computationally tractable form. This is often a challenge because a substantial portion of neurobiological data is qualitative in nature. **(2)** A model must be assembled from this raw data, which involves critical decisions on the appropriate level of abstraction and desired scope. **(3)** The model is typically simulated, either directly in the model construction tool or in a separate environment. **(4)** Finally, the simulation data must be analyzed, which is often non-trivial due to the potential scale of models today. Each of these four stages is unique, often requiring distinct forms of insight and benefiting from different aspects of expertise on the part of the user.

There are numerous software applications available for parts of one or several of these stages, some of which have been optimized over decades (Table 1). In particular, the simulation of neural systems **(step 3)** has benefited greatly from tools such as NEURON and GENESIS/MOOSE which facilitate the representation and simulation of complex neuronal dynamics and morphologies (Hines and Carnevale, 1997; Bower and Beeman, 1998; Dudani et al., 2009). Recently introduced simulators such as Brian and NEST have focused more on network simulations, and similar capabilities have been added to NEURON and GENESIS (Gewaltig and Diesmann, 2007; Goodman and Brette, 2008). Many of these network simulators have been parallelized to run on supercomputers. In general, simulators require the user to describe models in a programming language. Notably, some simulators, such as NEURON and GENESIS, also provide an integrated modeling environment that facilitates the user's work at various steps in the process, such as editing models and managing simulations. Having a programming language such as Python or C at the foundation of a neural modeling tool is greatly enabling for its functionality, as in theory these languages are both agnostic to scale or complexity.

**Table 1:**
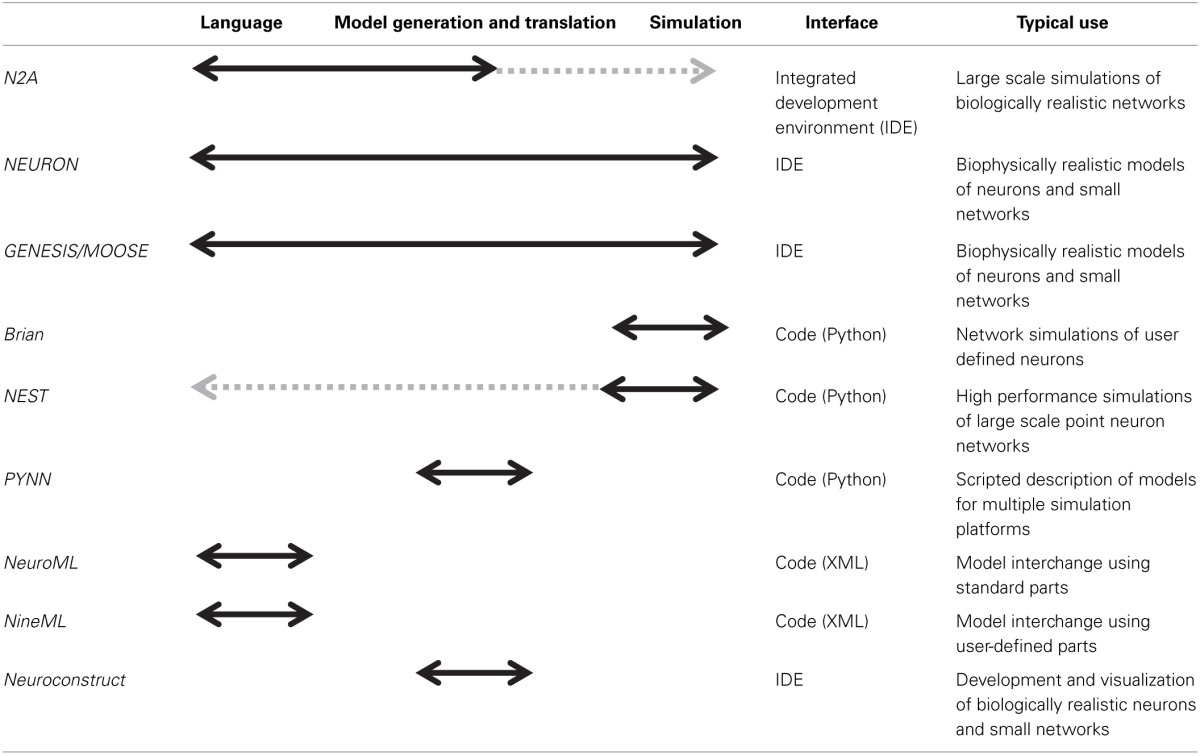
**Overview of different neural modeling tools**.

Primary scope is illustrated by bold arrows, with limited capabilities shown by shaded arrows.

Nevertheless, despite this plethora of tools, modeling neural systems is becoming ever more challenging, particularly as available computing resources and available biological data approach previously unimaginable heights. This trend toward incorporating more biological detail into models and integrative community efforts has led to the development of XML based descriptions such as NeuroML and NineML (Gleeson et al., 2010, 2011; Raikov, 2010) and model generation tools such as PyNN and NeuroConstruct (Gleeson et al., 2007; Davison et al., 2008) that are moving the community beyond stand-alone platforms toward model-sharing. Nonetheless, the use of standard parts, which is useful for model interchange, can be limiting when building models with complex features, such as structural plasticity or non-standard dynamics. Relying on a formal coding interface to go beyond pre-packaged modeling components often presents a challenge to the typical neuroscientist user. We expect this need to be particularly notable when a prospective modeler faces challenges such as structural plasticity (often important for clinical models), uncertainty quantification (necessary for any model with numerous free parameters), and parallelization of large-scale simulations. These are general problems with solutions that are often specific to a given network. For instance, while some network architectures map well onto GPUs (Richert et al., 2011), other networks map better to different system architectures.

Here, we present a new tool, *Neurons to Algorithms*, or *N2A*, which complements these existing approaches. Rather than focus on the simulation aspects, which are often specialized to the type of model being computed, we focus on the first two stages of modeling, the computational representation of neurobiological data (e.g., describing the projection pattern from DG to CA3 as a narrow Gaussian with sparse connection probability) and the descriptions of models themselves (Figure 1B). In this respect, it is most similar to PyNN, though with several important differences. First, N2A represents information in a flexible computable format that permits almost any neurological dynamics; whereas PyNN is more specialized to use canonical standards or native models represented within lower-level simulators. Second, N2A's hierarchical and relational design is inherently scale agnostic, forming a computable database for neural data. Finally, the part-relationship representation is suitable for both standard dynamical simulations as well as higher level network analysis.

We have designed N2A to be general in how it represents models, so we expect that it will be suitable for a wide range of neural modeling approaches. However, we recognize that some tools are exceptional in certain application areas (i.e., biophysical single neuron multi-compartment models in NEURON), and we expect those to remain the tools of choice in those domains and will seek to integrate N2A with their existing functionality. Rather, we believe that N2A will provide differentiating capabilities in high fidelity, large scale network models. These models have several key characteristics, including many distinct neuron and connection types, non-trivial connectivity patterns and part-to-part variability, and large parameter spaces with at times poor biological constraints that will require considerable sensitivity analysis and parameter exploration. This type of high-detail modeling is relatively new to neuroscience and is an increasingly common approach, enabled in large part by modern computing resources and the advances in high density physiology and anatomical data acquisition (Izhikevich and Edelman, 2008; Aimone et al., 2009; Richert et al., 2011; Markram, 2012) and by the recognition that therapeutic models will require consideration of the complexity of neural dynamics (Aimone and Weick, 2013).

## Overview of N2A framework

The translation from raw biological information into a model suitable for simulation is a non-trivial process. We recognized that a systematic approach capable of model development would require a structured language, a dedicated software platform, and use of community resources. Along these lines, the overall N2A framework we describe here has three significant components: the N2A language, the N2A software, and integration into the broader community. First, we will introduce the N2A language, which is our approach for describing neural models that enables the description of neural data in a computable format from which models can be constructed. Second, we will describe the current N2A software application, which includes both a user interface and a custom database. Third, we will discuss our vision for how N2A fits into the broader neuroscience community, which includes both the integration of N2A into existing neuroinformatics frameworks and collaborative N2A peer-to-peer networks.

The N2A tool is open source and is available at http://code.google.com/p/n2a.

## Model description language

The N2A language was designed with the primary goal of being capable of representing as much neural data as possible in a simple computable format. In this context, computable refers to the ability for an observer, whether a human or a machine, to read the description and integrate it into a simulation. A simple rule-of-thumb is that for a model to use neural information, it either has to be represented by an equation or in the structure of the model. For some classes of neural data, such as the behavior of ion channels and membrane voltage dynamics often characterized in electrophysiology studies, representation in a computable format is as simple as writing the canonical differential equations (see HH example below). For other types of data, computability is less straightforward; for instance describing the dynamics of dendrite growth will likely be a non-trivial pursuit involving approaches such as L-Neuron (Ascoli and Krichmar, 2000). N2A refers to units with largely self-contained dynamics (e.g., a neuron or a dendritic spine) as *parts* and the equations governing its dynamics as its *equation set*.

**Figure 2:**
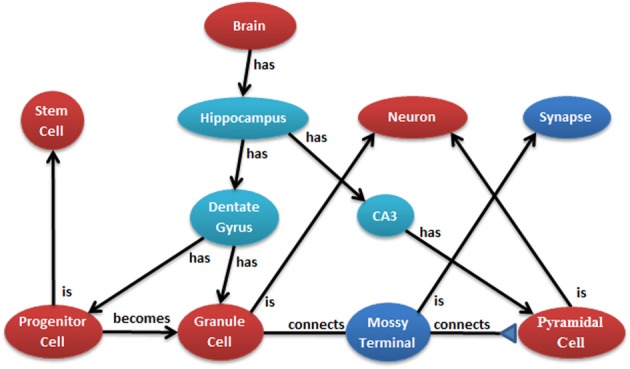
**Example of hierarchical relationship of a neural system that can be mapped into N2A.** N2A uses a parts/connections framework to describe a model's components and how they interact. Further, parts and connections can inherit dynamics and relationships from parent parts (e.g., a granule cell is a neuron) that allow the models described within N2A to be related to neurobiological data characterized within neuroinformatics ontologies.

The conversion of neural anatomy information into a model's structure is a major goal of N2A which is best illustrated by an example. Figure 2 illustrates a few cell types in the hippocampus from one common point of view. Ontologies, such as those at NeuroLex and Open Source Brain (Gleeson et al., 2012; Larson and Martone, 2013), describe the parts and relationships of a system. Each object in the ontology can have any number of attributes, and an important job of the ontology is to provide consistent naming of those attributes across the entire community. Attributes may contain any kind of data, from a single number to text to an entire data series captured by a physiological experiment.

Examples of attributes might be:

Name = Hippocampus CA3 pyramidal call
Organism = Vertebrata
Neurotransmitter released = Glutamate
Dendrite Length = 12481.9 ± 2998.9 um

Most attributes can be thought of as a simple pair: attribute = value. N2A takes this one step further by representing the dynamics of a part as a set of equations. The attributes are the variables, and the values describe how those variables evolve over time. Equations describe how attributes interact with each other in an explicit computable manner. Such a mathematical representation can be incorporated into the metadata of any part in the ontology. The Examples section below shows what several models look like in practice.

### Part inheritance and inclusion

The N2A language specifies rules for how equation sets are combined which are motivated by object oriented principles from programming. When part **C** also *is a* part **P** (e.g., a granule cell *is* a neuron), part **C** *inherits* all the equations and metadata contained in **P**. **C** can inherit from any number of parents. A named value (equation or metadata) that is defined directly in **C** hides any value with the same name in a parent.

When a part **M** *has a* part **P** (e.g., the dentate gyrus *has* granule cells), a prefix is added to each equation from **P** as it is *included* into **M**. This allows the user to reference equations within included parts. N2A uses the full-stop character (.) to delimit prefixes. A value with **P**'s prefix that is defined directly in **M** hides any value in **P** with the same name, in much the same way that names in **M** hide names in **M**'s parents. **P** may in turn include a part **Q**, whose equations are all prefixed and placed in **P**. **M** can then hide any name in **Q** by using both prefixes. This can continue any number levels deep. For example, the brain model includes a hippocampus which includes a granule cell model. The brain model could contain an equation that specifically sets the number of granule cells in the population.

### Connections

To understand connections in N2A, it is important to recognize the difference between a part and an *instance* of that part. N2A distinguishes these notions in much the same way that an object-oriented language such as Java distinguishes between a class and an object. When a simulation runs, each part in the model can generate an entire population of instances, and each instance has its own distinct set of values for the state variables defined in the part. An equation set should be thought of as a template for stamping out instances.

A part **C** that *connects* two parts **X** and **Y** (e.g., the mossy terminal in Figure 2) is able to access their equation sets and make statements about how they couple to each other. **C** associates a prefix with each of **X** and **Y**, and uses those prefixes to access the respective variables. During a simulation, an instance of **C** may add to or otherwise modify values in the connected instances. **C** specifies rules about which members of population ***A*** to connect with which members of population ***B***. Instances of **C** are created or destroyed automatically as the populations grow and shrink.

### Structural dynamics

When an instance of part **P** *becomes* an instance of part **Q** (e.g., the progenitor cell *becomes* a granule cell in Figure 2), all values with matching names are copied into the new instance. **P** can split into any number of types, allowing one to model development and population dynamics. The N2A language commits to the notion that all morphology and connectivity are the consequence of the dynamics governing individual parts. These include rules for creating and destroying parts, splitting and changing type, and moving in space. The language provides a way to express all of these as equations.

### Scale independence

The N2A language is designed to model a system at a wide range of scales. Gene regulatory networks can be represented either as coupled parts or as a collection of state variables within a given part. Common protein interaction sequences, such as the MAPK pathway, can be represented as a part that is included in many other structures.

**Figure 3:**
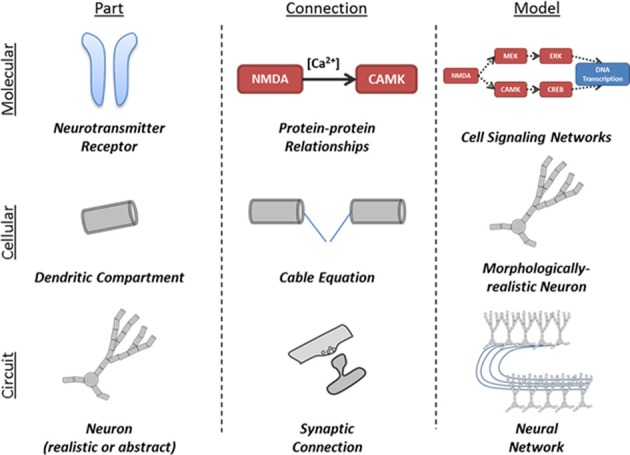
**Illustration of application of part-connection-model framework to different scales of neural simulations.** The structure of the N2A language allows it to be applied in scales ranging from molecular kinetics models of cell signaling to neural network models comprised of complex neurons and synapses.

The interaction of neuron populations is illustrated in Figure 2. Entire brain regions can be wrapped into parts and connected with each other. Each level of model can be represented by either a simple or a detailed part, allowing successive abstraction as one studies a system (Figure 3).

## Software

The N2A software attempts to ease many of the obstacles that researchers face while developing, executing and fine-tuning physiological models. To this end the software embodies these basic principles: transparency, traceability, repeatability, and sharing.

**Figure 4:**
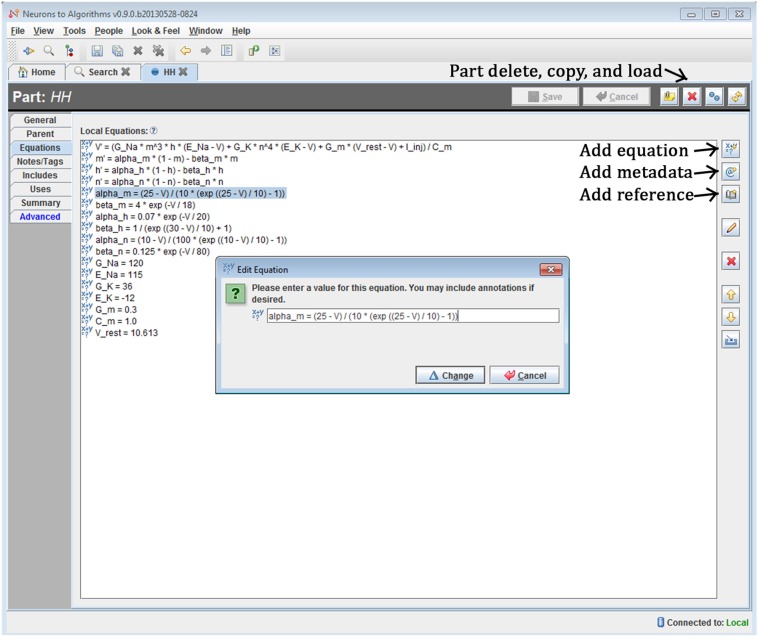
**Screenshot of N2A User Interface.** The N2A application allows users to create and edit equation sets for parts, define how they connect within a model, and incorporate metadata and references regarding literature sources into the model. Equations are input in a straightforward mathematical notation, with differential equations written using the “X'=” notation and constants defined directly.

**Figure 5:**
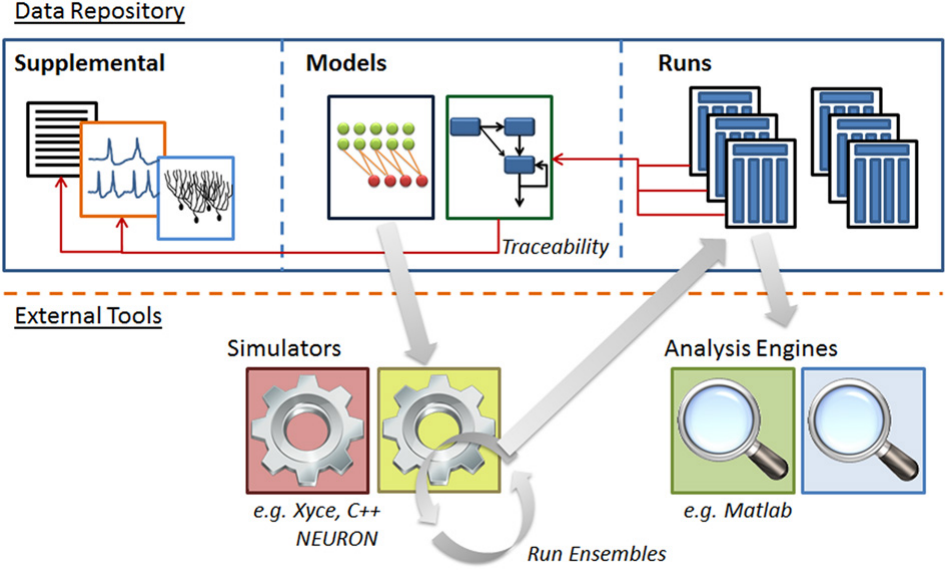
**Structure of N2A software database.** N2A stores three types of information within the database. (1) Raw data such as references and parameter fits that helps define parts' behavior (left), (2) model descriptions which consist of a set of parts and connections and information concerning their initialization and inputs (center), and (3) ensembles of runs that include model configuration, runtime metadata and results (right). N2A uses external simulation tools and analytical environments, though it does include a reference simulator implemented in C++ (bottom).

The system is a Java-based desktop application (Figure 4) with an embedded database (Figure 5). The interface provides the user with a method to locate models and other supplemental records, modify models, and create new sets of simulations (“run ensembles”) against a given model. Supplemental records could be references to papers, associated lab results, input data, or other related information that you want to track alongside the models. The user interface provides context sensitive help. It shows part hierarchies along with associated equation sets, metadata and references.

To support repeatability, the N2A software stores all model runs. A planned part of the design is to keep a version history for models (see below), so researchers can make changes without affecting the equations used in a previous simulation. Currently, run ensembles and individual runs maintain all parameter information in addition to their associated model. Run results are stored separately for analysis. Post-run/analysis products can potentially be tracked by the software given the right plug-in support. By recording every aspect of the model creation and execution, including system-generated random numbers and seeds, we enable repeatability for quality assurance and double-checking purposes.

No single tool can serve all purposes, so N2A is built from the ground up with extensibility in mind. The software uses a plug-in infrastructure to allow others to extend the product to meet their needs. A key class of extension is the handler (“backend”) for a given simulator, and a simple interface is provided for creating new ones. Additionally, new types of model and supplemental records can be added and visualized in the user interface according to the plug-in designer's wishes.

## Simulation capabilities

N2A is a model description language, but to make it useful in practice the tool is able to translate models into inputs to several different simulators. Each simulator is handled by a separate “backend” module. Currently, N2A has backend modules for two simulators: C++ and Xyce. To be fully useful it will need additional backends to support commonly used simulators such as NEURON, GENESIS, Brian, or NEST, and common middleware such as PyNN. As N2A becomes integrated with evolving neuroinformatics standards such as NeuroML, we hope to leverage multiple additional simulators. This is a key part of future work.

### C++

The C-backend is the reference implementation of the N2A language. It is capable of simulating any construct expressible in the language, including structural dynamics. The price for such generality is a loss of efficiency in specialized cases. For example, the C-backend is primarily designed for a general dynamical system, so it is less efficient on large spiking networks. The C-backend works by translating the model into a set of C++ classes, which are then coupled with a runtime library that handles object management and numerical integration. The entire simulation is a self-contained executable program.

### Xyce

Xyce is a parallelized version of the electrical circuit simulator SPICE that is capable of natively simulating large scale circuits on supercomputers. Recently, we have extended its capabilities for very large scale simulations of neural networks (Schiek et al., 2012). In addition to its traditional devices (transistors, capacitors, etc.), Xyce now also has neuron and synapse “devices.” Xyce parses and solves a broad range of explicit mathematical expressions, so model dynamics not covered by built-in devices can also be included. Currently, N2A is capable of translating most of its neural models into Xyce simulations, through a combination of direct equations and specialized neural devices.

## Future capabilities: model sharing and integration into the neuroinformatics community

The N2A tool is still under development, and the methods of sharing described in this section are aspirational, but high-priority future work. We summarize existing and projected capabilities in Table 2.

**Table 2:** **Status of current and future features of N2A**.

**Feature**	**Status**
**LANGUAGE SPECIFICATION**	
*Part and connection descriptions and inheritance*	Documented and implemented in N2A tool
*Structural dynamics*	Documented and implemented in N2A tool, limited backend support
*Composition of models as parts in other models*	Documented with some backend support. Tool allows composition of parts, but currently treats models distinctly
**N2A SOFTWARE**	
*Model/part search, metadata, reference documentation, version control*	Implemented in N2A tool
*Uncertainty quantification/sensitivity analysis*	Tool drives multiple simulations with parameter variation using different standard approaches
*Analysis of simulation results*	Not implemented; user must export to other tool (e.g., Matlab or Excel)
*Peer to peer communication*	Not yet implemented
*Exchange models with community*	Not yet implemented. Plan to add NeuroML import/export
*Visual editing of network structure*	Partially implemented
**SIMULATION BACKENDS**	
*C++ Backend (reference implementation)*	Implements most N2A language specifications. Structural plasticity only partially implemented
*Xyce*	Implements dynamical equations directly; implements event-driven synapses through pre-built devices
*Other simulators*	Future intent to develop export capability to other tools

Ideally, all models associated with a given part should be stored in a central repository accessible to everyone, such as the Neuroscience Information Framework (NIF) or Open Source Brain (Gleeson et al., 2012). NIF is particularly compatible with our vision because they organize all data according to the NeuroLex ontology and they offer curation for small quantities of data. Since N2A models are very concise they fit into this category.

**Figure 6:**
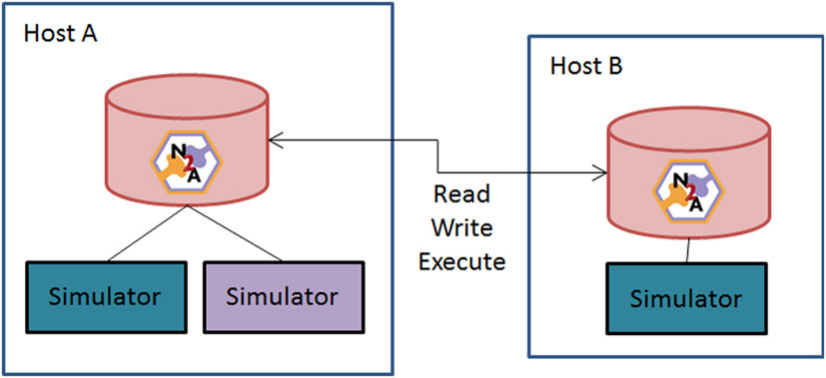
**Schematic of N2A peer-to-peer collaborative community vision.** N2A can be run with either a local database or through a common network repository. Users will be able to directly share models between collaborators or with the broader community through opening their database to the broader neuroinformatics community.

Figure 6 illustrates a second means of sharing. A user asks the N2A tool to act as a server online and allow peers to access data and compute resources. This *Peer-to-Peer (P2P)* arrangement brings up two closely related issues: versioning of models and the repeatability of simulations. The problem is this: if a researcher configures a model a certain way, simulates it, and later some part that the model depends on is changed, it is no longer possible to produce exactly the same simulation again.

We propose to keep all parts/models under *version control*. Examples of version control systems in the software-development world include Subversion, Git, Mercurial, etc. An N2A data-store would not directly use these tools, but instead implement similar concepts. Any time a model is transmitted between two peers or simulated, a version is permanently recorded in the database. All parts it depends on are also permanently versioned. Ongoing development of a part goes into a subsequent version, and does not have any influence on the content of a model. To ensure repeatability of simulations, it is necessary to record a number of details beyond the model itself, such as the simulator used, random number seeds, platform, etc. It may not be possible to capture every detail and make a simulation perfectly repeatable, but a record of the key variables will help in interpreting the results of the experiment.

To further drive integration into the neuroinformatics landscape, we envision that the N2A tool will be compatible with existing tools by leveraging the increasingly common standards for model definition, such as NeuroML, LEMS, and NineML. As other simulation frameworks and environments specializing in other classes of neural simulations develop support these growing standards, we expect that linking the models defined within N2A into those simulation environments to be relatively straightforward.

## Examples

Here, we show three different examples of the neuroscience systems implemented within the N2A tool to illustrate how it represents progressively more sophisticated neural circuits. These are not a complete sample of N2A's applicability; rather these examples are intended to highlight the scope of N2A and its eventual vision.

## HH model

The Hodgkin-Huxley (HH) model of spike generation and propagation underlies many computational modeling studies and is well suited to illustrate how N2A represents neural dynamics (Hodgkin and Huxley, 1952). Briefly, the Na+/K+ ion channel version of the HH model is a system of four differential equations with two state variables governing the dynamics of Na+ ion channels (*m* and *h*), one state variable governing dynamics of K+ ion channels (*n*) and a state variable (*V*) representing the internal voltage of the neuron or axon. *V* is often represented by the equation

$$CV^{\prime }=g_{Na}m^{3}h\text{​}\left(E_{Na}-V\right)+g_{K}n^{4}\text{​}\left(E_{K}-V\right)+\;g_{\text{leak}}\text{​}\left(E_{\text{leak}}-V\right)+I$$

where *C* is membrane capacitance; *g*_*Na*_, *g*_*K*_, and *g*_leak_ are maximum conductances for Na+, K+ and leak currents, respectively; *E*_*Na*_, *E*_*K*_, and *E*_leak_ are the reversal potentials for those respective currents; and *I* is input current. The state variables *m*, *n*, and *h* typically take the form

$$x^{\prime }=\alpha_{\text{x}}\text{​}\left(V\right)\left(1-x\right)-\beta_{\text{x}}\text{​}\left(V\right)x$$

where α_*x*_(*V*) and β_*x*_(*V*) are functions of voltage specific to each state variable.

### N2A representation

**Figure 7:**
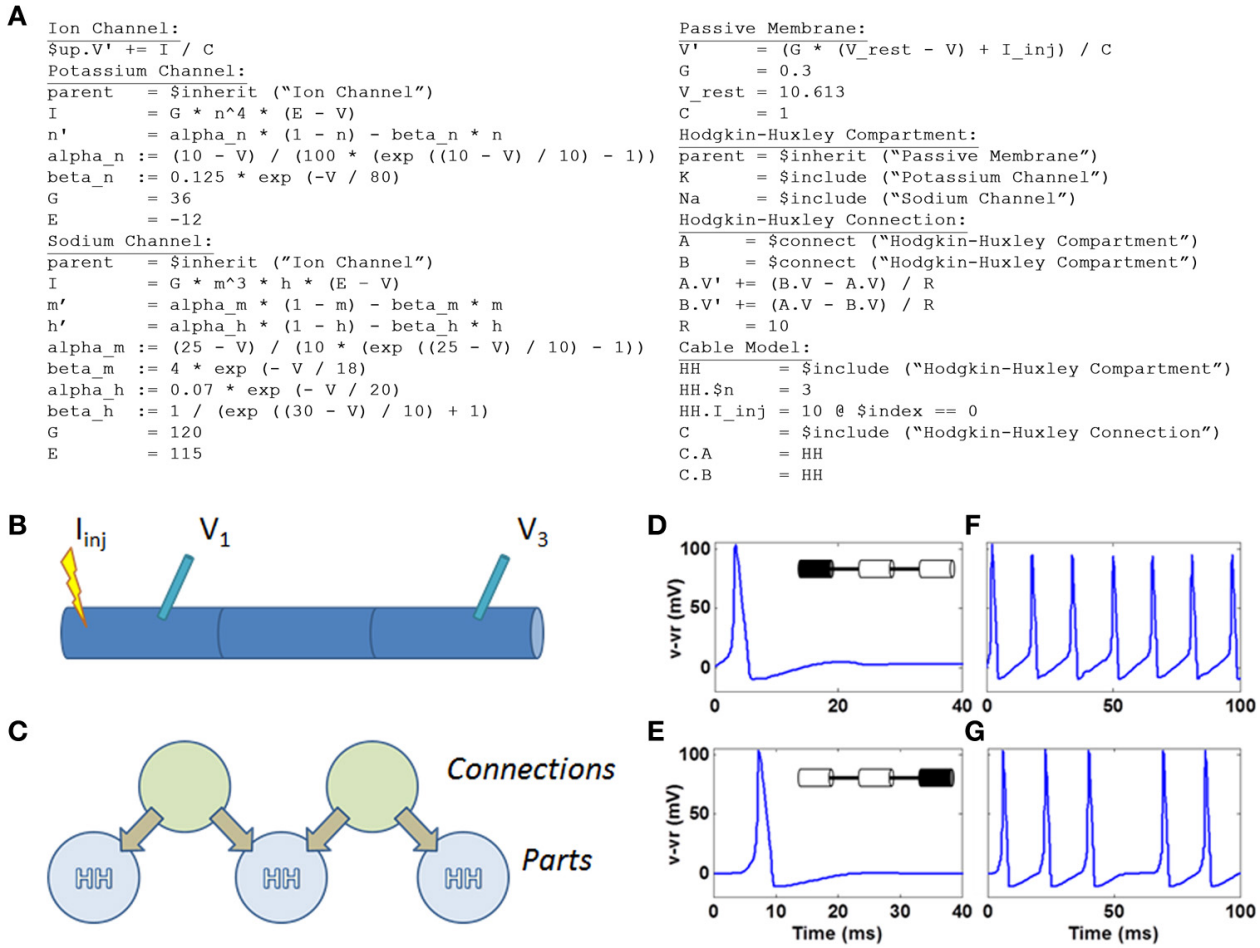
**HH model defined within N2A and simulated on Xyce. (A)** N2A language depiction of HH dynamics **(B)** Cartoon illustration of 3 compartment HH cable. Current is injected into left compartment and measured in left and right compartments **(C)** N2A representation of HH cable. **(D)** Action potential in left compartment (site of current injection). **(E)** Propagating action potential in right compartment. **(F)** Spike train in left compartment in response to persistent current. **(G)** Propagating spike train in right compartment.

Within N2A, we represented the HH model using the equations outlined in (Koch, 2004) in a simple 3-segment cable configuration (Figure 7). While N2A can represent the HH dynamics of individual compartments using a part that contains all of the equations for the sodium, potassium, and leak currents, we chose to construct the demonstration model as a part with only passive membrane dynamics that “includes” the appropriate ion channels, in this case Na+ and K+. This separation of ion channels from host compartments facilitates the reuse of well-tuned ion channels in multiple independent neuron models as well as the rapid interchange of one ion channel to another within a given model. Each of the three HH compartments are coupled by a simple connection part that implements the cable equation

$$\begin{array}{l} A.V^{\prime }=g_{r}\text{​}\left(B.V-A.V\right)\\ B.V^{\prime }=g_{r}\text{​}\left(A.V-B.V\right) \end{array}$$

where *A.V* and *B.V* are the voltages of the two connected HH compartments and *g_r_* is the lateral membrane conductance.

Below is the complete set of equations expressed in the N2A language (Figure 7A). This example contains seven parts: the abstract ion channel, two ion channels that inherit from it, the abstract passive compartment, the HH compartment that inherits from it and includes the two ion channels, the HH connection, and finally the model that incorporates the HH compartment and HH connection into a cable. For a more thorough explanation of how the language expresses this model, see the “N2A Language Overview” in the supplementary material.

We illustrated simple HH dynamics and propagation of action potentials by injecting 10pA into the left compartment (Figures 7B,C) and, in an effective current clamp condition, observed voltage deflection representing the 100 mV spiking event in the compartment (Figure 7D). The spike propagates to the right-most compartment with a short delay (Figure 7E). A longer current injection yields a series of spikes in the leftmost compartment (Figure 7F) that again is manifested two compartments away (Figure 7G), albeit at a short delay and with a notable failure to propagate of one spike.

## Sensitivity analysis of balanced excitation/inhibition networks

Balanced excitation/inhibition (E-I) networks have attracted attention as a coarse model of cortical dynamics (Vogels and Abbott, 2005; Brette et al., 2007). Often containing a mixture of 80% excitatory and 20% inhibitory spiking neurons (though studied with both other ratios and in non-spiking systems), E-I networks can show a range of non-trivial “phases” of dynamical network activity, including oscillatory and chaotic (or near-chaotic) behaviors. Balanced E-I models are interesting for a number of reasons, among which is their increasing relevance in understanding motor and prefrontal cortex dynamics and their relationship to the reservoir computing research area in machine learning. Specifically, it appears that the chaotic dynamics observed under certain conditions are computationally uniquely powerful (Laje and Buonomano, 2013).

Clearly, not all configurations will produce complex chaotic or near-chaotic behavior; indeed understanding the effects of design and parameters on these dynamics is an active area of research (Litwin-Kumar and Doiron, 2012). Here, we illustrate the parameter exploration capabilities of the N2A tool by systematically varying two basic parameters that affect the behavior phase: strength of recurrent excitation (E) and strength of recurrent inhibition (I).

### N2A representation

**Figure 8:**
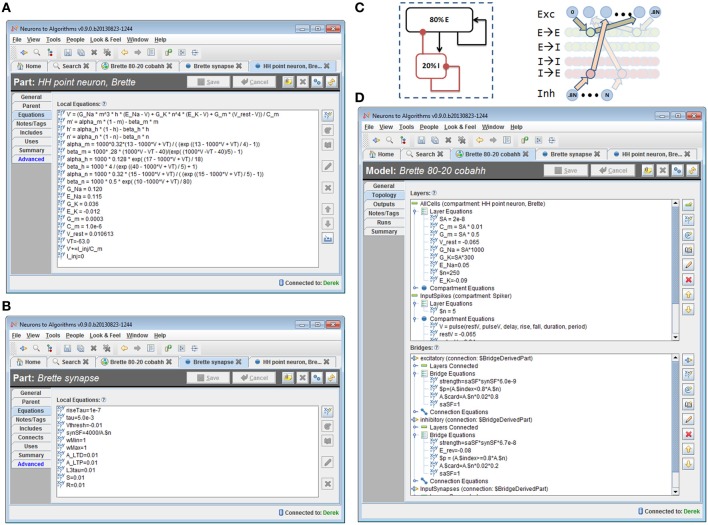
**80/20 E-I network definition in N2A. (A)** Screenshot of part equation set for spiking neurons used in 80/20 model. **(B)** Screenshot of synapse equation set for connections between neurons in 80/20 model. **(C)** Simple network illustration of 80/20 model from a block perspective (left) and instantiation perspective (right). **(D)** Screenshot of model definition equation set for 80/20 network in N2A; differences in excitatory and inhibitory connections (namely conductance and reversal potential) as well as the sparse inputs are defined at this level.

**Figure 9:**
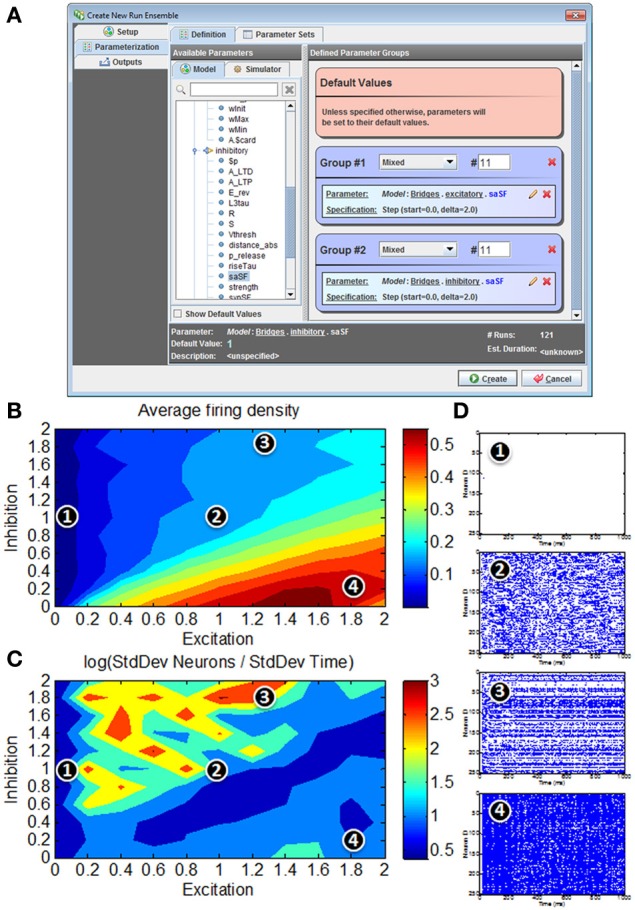
**Model simulations of 80/20 E-I network in N2A. (A)** Screenshot of parameter search window within N2A; any model parameter can be varied either randomly or systematically and in isolation or in conjunction with other parameters. **(B)** Firing rates in response to parameter sweep of differing E and I synaptic conductance levels. **(C)** Distribution of activity in response to parameter sweep. **(D)** Representative raster plots of spiking from E-I network at different positions in parameter state space; 1—all neurons effectively silent; 2—asynchronous firing dynamics; 3—skewed firing dynamics, with subset of neurons exhibiting persistent activity; 4—hyperactive network with most neurons persistently active.

We implemented the model described in benchmark 3 of (Brette et al., 2007) in N2A, then used N2A to define and execute a “run ensemble” of 121 simulations with different values of synaptic conductance (Figures 8, 9). Building the 80-20 network model consisted of creating the necessary parts, defining cell populations (“Layers”), and defining connections between cells (“Bridges”) both within and across populations. The N2A parts used for this model were: (1) a variant of a Hodgkin-Huxley neuron described in the Brette paper (Figure 8A) (2) a conductance-based synapse also described in the Brette paper (Figure 8B), (3) an artificial “Spiker” neuron to provide input into the network, and (4) another exponential synapse to connect the “Spiker” cells to selected cells in the main population. Xyce has built-in implementations of the Brette neuron and synapse models, so the N2A parts included metadata indicating that those implementations should be used. Figures 8C,D shows how populations and connections are identified in N2A. All neurons in the 80-20 network have the same dynamics, so we created a single population of neurons using the same N2A part, but made excitatory connections only to the first 80% by index. Excitatory and inhibitory connections used the same “Brette synapse” part. We used connection equations both to override part parameter values as appropriate for excitatory or inhibitory connections and to specify which neurons can be connected.

The “Runs” tab shown in Figure 9A allows the user to create and run one or more simulations of the model. Any parameter defined in the model can be dragged from a pre-populated list into the run ensemble definition, with search strategies ranging from simple step protocols to Monte Carlo and Latin Hypercube sampling. The figure below shows selection of the two synaptic conductance coefficients varied to produce Figures 9B–D, the number of values for each and how they varied. In this case we simply stepped through a range of values at fixed intervals. Certain simulation parameters such as seed or integration method can be chosen or varied in the same way.

Unsurprisingly, for low E the network exhibits very low average firing rates, whereas high E with low I yields very high average firing rates (Figure 9B). For roughly balanced E and I levels, the overall firing rates appear to be comparable in spite of absolute magnitude. However, a simple measure of activity distribution (Figure 9C) shows that even for E, I combinations with comparable firing rates, the dynamical state of the network can differ considerably; suggesting that there are at least four clear states of network activity observable in our small search space (it should be noted that it is not surprising that high-dimensional networks such as these can exhibit many different phases of behavior). Figure 9D shows representative examples of network activity at different positions in the parameter space.

It is interesting to note that even this simple illustration of the parameter searching capabilities of the N2A tool provides results that merit more detailed exploration. It was not surprising that these networks not only exhibit silent (**1**) and hyperactive (**4**) states in addition to the originally published asynchronous state (**2**), but we did not expect this simple parameter exploration exercise to show a state where the network activity is preferentially localized to a subset of highly active neurons (**3**). What is not clear from this study (or indeed many other studies of these abstract networks) is how these dynamics relate to real *in vivo* cortical function. For instance, it has been suggested that working memory in the pre-frontal cortex (and other cortical areas) involves a switch from asynchronous activity to a more persistent activity of a subset of neurons holding a trace (Durstewitz et al., 2000; Wang, 2001). These illustrative parameter search results are far too preliminary to make any strong links to this neurobiological phenomenon, however it would be interesting to expand the search to include the both more realistic network connectivity (Litwin-Kumar and Doiron, 2012)and extrinsic neuromodulatory influences such as dopamine(Brunel and Wang, 2001) that may effectively alter the excitation/inhibition balance dynamically.

## Structural dynamics

In addition to challenges in understanding parameter sensitivity of models, many neural systems involve dynamics or structures that are not well suited to existing tools. One such example is structural plasticity of neural systems. While most modeling studies treat neural circuits as effectively fixed, at most implementing plasticity in synaptic weights, there are many neural processes that necessitate changing the network itself over extended time scales. These include neurological and psychiatric disorders, development, and even structural plasticity in the healthy adult brain through neurogenesis and dendritic spine dynamics.

### N2A representation

The structure of parts within N2A allows for the representation of the regulated birth, transitions, and death of instances. Here, we show two examples of how structural plasticity would be represented within the N2A language. There are two key language commands: assigning **type** to an instance of a part will transition it to a different type of part (i.e., differentiation), and assigning multiple types to an instantiated part will replicate the instance. This division can either be symmetric (where both children are equivalent to one another, regardless of whether the parent's type is maintained) or asymmetric (where the children are different parts, with one perhaps retaining the parent's type).

**Figure 10:**
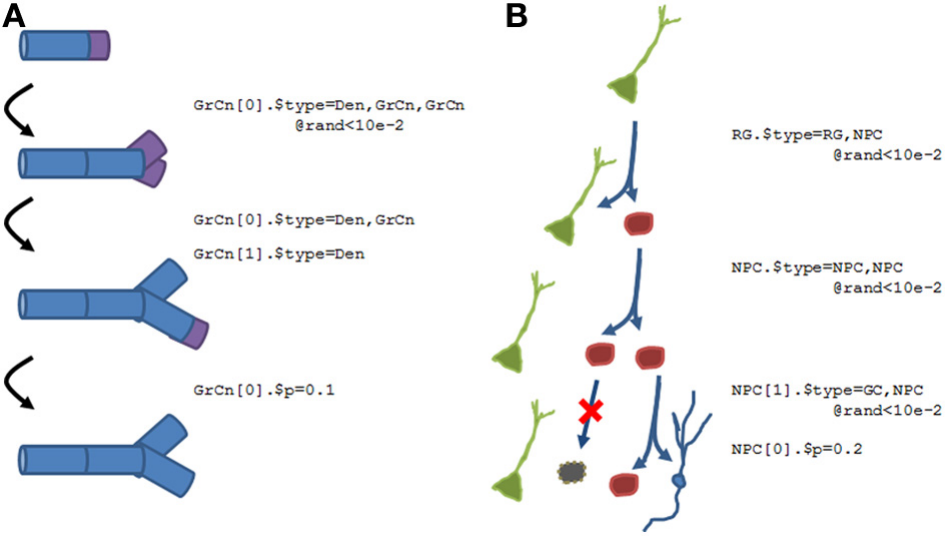
**Examples of how structural plasticity would be expressed within N2A. (A)** Growing dendrite with growth cones. Progressive series of growth cone (GrCn) branching, differentiation, and death can lead to the construction of a complex arborization. Each step of this process could be regulated by other variables, such as external chemical signals or intrinsic activity. **(B)** Proliferation and Differentiation from a stem cell population. A radial glial cell (RG, green) can divide asymmetrically, producing a copy of itself and a neural progenitor cell (NPC, red). This NPC can then itself divide symmetrically, asymmetrically, differentiate, or die. As with the growing dendrite, each of these events can simply be probabilistic or regulated by other factors.

Figure 10 shows two examples of what the N2A language will accommodate. Figure 10A shows a growing dendrite, with a dynamic growth cone (purple) at the end. This growth cone is capable of linear growth (basically splitting into an ordinary compartment and the growth cone), branching (splitting into multiple growth cones and a stable compartment), differentiation (growth cone becomes a compartment), or death (growth cone simply disappears).

Figure 10B illustrates a different form of structural dynamics, the proliferation and differentiation of a dentate gyrus stem cell into an eventual granule cell population during the adult neurogenesis process. In this example, a radial glial cell (RGC, green), which is considered the primordial stem cell type in the adult dentate gyrus, is capable of asymmetric division, producing a neural progenitor cell (NPC, red) as well as a “copy” of itself. This NPC subsequently exhibits several rounds of symmetric cell division, amplifying the number of children. Finally, the NPCs will either die (black) or differentiate into granule cells (blue).

## Summary

N2A has been designed to enable the general neuroscientist to achieve the scope and depth of models that heretofore have been mostly limited to those with considerable programming expertise. Concepts such as structural plasticity and parameter searching that are illustrated can all be achieved using other tools or conventional languages, but they often require considerable work on the modeler's part. We believe that the trends in neuroscience toward more detailed characterization of systems and increased emphasis on clinical conditions (such as diseases and therapeutic mechanisms) will further amplify the importance of having a tool to effectively capture neurobiological complexity in a straightforward manner.

The increase in high throughput data acquisition, improved neuroinformatics tools, and growing availability of computing resources all will facilitate the trend toward more biologically detailed approaches to modeling neural systems. An important consideration is that the rationale behind biologically realistic models is quite different than that of other modeling approaches, such as large scale simple models and abstract models of neural system functions. Briefly, in contrast with models that illustrate how a neural circuit can map to a known function, “bottom up” detail oriented models can suggest novel computational functions for neural processes that otherwise would not have been considered. Such work has in the past been useful in identifying the functions of complex neural processes; for instance a high resolution model of neurogenesis was able to suggest that new neurons may provide a previously unknown function of encoding time into episodic memories (Aimone et al., 2009), a function that has subsequently been measured in rats (Morris et al., 2013).

Notably, the potential value of “bottom up” models in providing novel functional insight into a brain region is dependent on a design of the model that is not biased toward desired results. The brain is, of course, considerably more complex than any single model is capable of representing, and abstraction is thus always necessary to some extent. However, abstraction should be performed with careful consideration to minimize disruption to behavior, not simply guided by the ease of implementation or the ready availability of data. The N2A tool is well suited for this challenge; as its representation of neuronal dynamics enables the incorporation of complex processes that are often neglected into models, such as adult neurogenesis or cellular protein kinetics. Furthermore, we believe that as N2A is further integrated into the broader neuroinformatics community, modeling biases due to the local availability of information will be minimized (e.g., someone may include the oft-ignored CA2 region in a hippocampal model if N2A can pre-populate the relevant details).

We recognize that N2A's data-centric, dynamical representation of neural information makes it less well suited for other modeling approaches, for which we expect many existing tools to be preferable. This includes Monte Carlo type simulations of molecular dynamics (e.g., MCell) and morphologically defined models of dendritic dynamics (e.g., NEURON). Notably, the original motivation of N2A was to automatically extract computational structures (the “algorithms” in the name) from data about neurons and their interconnections. Although the goal of automatic model reduction for algorithm discovery is now considered remote, its influence lingers in the design of the language and tools. For example, an increasing fraction of data in neuroscience exists in databases and is machine readable and computable, incorporating both graphical structures and dynamics.

The long-term goal of understanding the computation of the entire brain appears in the community sharing and neuroinformatic aspects of the tool. It is necessary, after all, to have a *computational* framework capable of representing the entire nervous system. In that sense N2A shares aspirations with cognitive frameworks such as ACT-R and SOAR, but it makes far fewer commitments to specific structure. Rather the expectation is that a large community of experts will jointly assemble what they know onto the scaffolding to create a digital mind. Undoubtedly the current incarnation of the language will evolve many times and perhaps even go extinct before the community reaches that goal.

## Conflict of interest statement

The authors declare that the research was conducted in the absence of any commercial or financial relationships that could be construed as a potential conflict of interest.
