# Supplementary material for: N2A: a computational tool for modeling from neurons to algorithms
Source: Front Neural Circuits. 2014 Jan 24;8:1. doi: 10.3389/fncir.2014.00001 (PMC3901007; doi:10.3389/fncir.2014.00001)
Supplement: Supplementary file 1 [file DataSheet1.DOCX]

**N2A Language Overview**

This is an informal presentation of the Neurons to Algorithms (N2A) modeling language. It is intended to give users an understanding of the behavior they can expect from models they create.

**General Concepts**

A “model” is any object described by a collection of metadata and equations, and which has relationships with other objects in the collection. A “part” is a model that has been included into another model. (See below for the distinction between inclusion and inheritance.) During a simulation, the top-level model may also be thought of as a part that is held by the simulator.

A part can either be a “compartment” or a “connection”. A compartment may be an entire neuron, a segment of a neuron, or any other kind of system component one may wish to model. A connection may be a synapse between two neurons, a shared surface between two segments of a single neuron, or any interaction between two system components that one wishes to model.

A given part may produce any number of instances within a system. Each instance has a separate and independent set of state variables, which evolve according to the dynamics prescribed by the part’s equations. A part is a template for stamping out instances. Sometimes this document conflates a part and an instance of that part, but keep in mind that all actual operations must occur on instances during simulation.

Each equation in a part declares a state variable via simple assignment:

<name> <assignment operator> <expression>

For example:

a = b + 10

This equation brings the state variable “a” into existence, and describes how it changes over time. In particular, it is always 10 greater than “b”.

Equations may be of roughly three basic types. A constant is known before execution starts and never changes. A regular variable receives its value from the expression, and generally changes during the simulation. A differential equation is like a regular variable, but implicitly creates an integrated value as well. Differential equations are always with respect to time. Example:

a = 10 // constant a

b = a * v // variable b

v’ = c * g // differential equation v’ and integrated value v

An equation set describes how the state of a part changes at a given instant in time. All equations are evaluated at the same moment, in parallel. This implies that there is no ordering among the equations. One equation cannot build on the results of another *at that instant in time*. (However, see “Multiline Equations” below for a relaxation of this rule.) For example:

a = c + 1

b = a + 1

c = b + 1

Before the first evaluation of this set, all three variables are initialized to zero. After the first evaluation, all three variables are 1. After the second evaluation, all three variables are 2, and so on.

The simulator determines how much time has transpired since the last evaluation. (That quantity is available in the special variable $dt.) Any differential equations are integrated over that interval, and the results are available at the moment the equation set is processed.

**Multiline Equations**

The rule is “one variable, one equation”. During a given update cycle, only one equation in a given equation set can change a variable. However, there are a number of useful expressions that *appear* to contradict this.

Append Operator – A connection modifies values within the parts it connects. This implies that a variable may be addressed by more than one equation set during a given update. The connection may simply assign a value, but since all evaluation is unordered this may or may not be the final value of the variable. Alternately, the connection could add some amount to the value that is already there using the append operator “+=”. (See “Combining Parts” below for an example using this operator.) Any variable touched by an append operator becomes a sum. A sum is set to 0 at the start of an update cycle and accumulates value from all equations that address it. Note in particular that the meaning of += is different from C-like programming languages. It *cannot* be used as a shortcut for “a = a + b”!

Conditional Evaluation – Sometimes an equation may take several different forms, depending on the state of other variables. Example:

sgn = 1 @ x > 0

sgn = -1 @ x < 0

sgn = 0

In this example, x is a state variable somewhere else in the equation set, and we are determining its sign.

The expression after the “@” is evaluated for each line that defines the same variable. If several are non-zero, then one will be chosen in a simulator-specific fashion. Examples of choice methods include picking a different one at random during each update, or always executing the first one. A user should *not* rely on any particular choice method. It is best to ensure that the conditions are mutually exclusive. A line with no @ is the default expression. If all @ expressions evaluate to zero, then the default is executed. If no default is provided, then nothing is executed. During init-time, a line with simply “@ $init” is treated as the default equation.

The reserved variable $init is set to 1 when a part is instantiated, and becomes 0 after its equation set is first evaluated. This allows initial values to be expressed as part of the equation set. Example:

V’ = g / C

V = -72 @ $init // V is integrated V’, except on first cycle

Another use of @ is to evaluate equations at specific places or times in the simulation. This can provide a simple way to create input patterns. Examples:

// Note: $t is current simulated time

a = 10 @ 0.9 < $t && $t < 1 // only during a certain period

b = 5 @ $xyz == [5;5;0] // only at a certain place

// Initialize c to 5, except at one place in the population.

// The first line is treated as the default during init,

// so the second line is given precedence.

c = 5 @ $init

c = 10 @ $init && $xyz == [2;1;0]

Temporaries – It is sometimes useful to define values that are not part of the state of a part. That is, you do not want to consume memory storing the value between updates. Instead you want to calculate each time for use in other expressions. Using the temporary assignment operator “:=” keeps a variable out of the part’s state. Example:

a := b + c

e = a + d // equivalent to b + c + d

g = a + f // equivalent to b + c + f

Temporaries are evaluated only when they are referenced by other equations. A temporary is only evaluated once per cycle, and the value is not stored between cycles. Temporaries must not form a cyclic dependency. Connections may not reference temporaries in other parts.

A simulator backend should automatically find sub-expressions within the equation set and factor them out to minimize computation. Thus it is not necessary to identify temporaries solely for the sake of optimization.

**Combining Parts**

A model may incorporate other models. When it does so, all the equations from the source model must be combined into a single namespace within the destination model. This may happen in two ways: inheritance and inclusion. These are defined below, followed by an extended example.

A = ...

B = ...

C = ...

Child / Destination

D = ...

E = ...

Parent / Source

When a model is *inherited*, all its equations are appended to the child model directly. If a variable is defined in both the parent and the child, then the child equation overrides the parent. If multiple models are inherited that define the same variable, and the child does not otherwise override it, one of the parent equations is chosen in an arbitrary way. The user should have no expectations about precedence. In the case of triangle inheritance (where C inherits from A and from B, both of which inherit from P), the equations from P will appear only once in C.

When a model is *included* as a part, a prefix is added to each variable defined in the part. This creates a sub-namespace that contains the equations from the part. The containing model may also define variables in that sub-namespace, and these override variables coming from the part.

If the included part references a variable but does not define it, then the containing part must provide it. The simulator will attempt to resolve a name in the local namespace first. If that fails, it will search in the containing namespace, and on up the hierarchy until the name is found.

In some cases, a variable may be defined in both the included set and the containing set. If you wish to force resolution up to the containing set, prepend the special namespace “$up” to the variable name. Alternately, you can explicitly prepend the containing namespace to the variable, but this is only possible when that namespace is known ahead of time. Some parts (such as ion channels) may be included in many different containers with many different names. The special namespace $up makes it possible to force resolution up the containment hierarchy without knowing those names.

A model can override one or all the conditional equations associated with a given variable in a part that is inherited or included. If the model contains an equation with the right prefix and variable name but no @, then all the equations associated with the variable are removed, and replaced by the new default equation. If the model contains an override with an @ clause, then only the equation with the matching @ clause (if any) is replaced. If a model specifies an empty @ clause, then only the default equation from the source part is overridden. Example

// In model “Bob”:

sgn = 1 @ x > 0

sgn = -1 @ x < 0

sgn = 0

// In model “Sue”:

B = $include (“Bob”)

B.sgn = 1 // no “@”; replaces all 3 forms of “sgn” with single equation

B.sgn = 1 @ // nothing after the “@”; replaces only sgn = 0

B.sgn = 22 @ x > 0 // replaces only sgn = 1 @ x > 0

Inheritance and inclusion make the construction of large models easy. The following describes what happens when you use inheritance and inclusion to assemble parts into a model.

A “population” is a set of part instances. The number of instances is specified by the reserved variable $n. (See the section “Model Layout” for details on the construction of a population.) A model may contain several different parts, and each part may define its own $n. This implies that there may be hierarchical production of populations. That is, each instance in a population may itself contain populations of subparts. The total number of instances of a particular part is the product of its $n and the $n of each containing part. If $n is not specified, then it defaults to 1.

Below is a fully-worked example of the Hodgkin-Huxley cable model. It starts by showing each source model with only their locally-defined equations. Then it expands the Hodgkin-Huxley Compartment equation set to show how inheritance and inclusion work. It concludes with the cable model and its fully expanded equation set to show how hierarchical inclusion works. Some models have brief commentary to highlight features.

Passive Membrane (a kind of compartment):

V' = (G * (V_rest - V) + I_inj) / C

G = 0.3

V_rest = 10.613

C = 1

Ion Channel:

$up.V' += I / C

The special namespace $up in front of V’ forces it to reference the enclosing part. I and C are not defined here; they don’t appear on the left-hand side of an equation. Therefore they must be satisfied by the enclosing or inheriting part.

Potassium Channel:

parent = $inherit (“Ion Channel”)

I = G * n^4 * (E - V)

n' = alpha_n * (1 – n) – beta_n * n

alpha_n := (10 - V) / (100 * (exp ((10 - V) / 10) - 1))

beta_n := 0.125 * exp (-V / 80)

G = 36

E = -12

“parent” is a pseudo-variable. It is not actually computed at simulation time. The exact name is arbitrary, but must be unique in the equation set. In particular, each $inherit() line must have a distinct name. This supports versioning of equation sets, where an $inherit() may be revoked or replaced.

When “Ion Channel” is inherited, $up is brought in verbatim. It is only resolved when this model is included as a part of another model.

Sodium Channel:

parent = $inherit (“Ion Channel”)

.V’ += I / C

I = G * m^3 * h * (E – V)

m’ = alpha_m * (1 – m) – beta_m * m

h’ = alpha_h * (1 – h) – beta_h * h

alpha_m := (25 – V) / (10 * (exp ((25 – V) / 10) – 1))

beta_m := 4 * exp (- V / 18)

alpha_h := 0.07 * exp (- V / 20)

beta_h := 1 / (exp ((30 – V) / 10) + 1)

G = 120

E = 115

Hodgkin-Huxley Compartment:

parent = $inherit (“Passive Membrane”)

K = $include (“Potassium Channel”)

Na = $include (“Sodium Channel”)

“K” and “Na” are pseudo-variables but they serve a crucial role: they specify the prefix for the included part.

Expanded equations for Hodgkin-Huxley Compartment: Note that variables on right-hand-side are fully qualified to show where they get resolved.

V’ = (G * (V_rest - V) + I_inj) / C

G = 0.3

V_rest = 10.613

C = 1

K..V’ += K.I / C // ..V’ refers to containing V’

K.I = K.G * K.n^4 * (K.E - V)

K.n’ = K.alpha_n * (1 – K.n) – K.beta_n * K.n

K.alpha_n := (10 - V) / (100 * (exp ((10 - V) / 10) - 1))

K.beta_n := 0.125 * exp (-V / 80)

K.G = 36

K.E = -12

Na..V’ += Na.I / C

Na.I = Na.G * Na.m^3 * Na.h * (Na.E – V)

Na.m’ = Na.alpha_m * (1 – Na.m) – Na.beta_m * Na.m

Na.h’ = Na.alpha_h * (1 – Na.h) – Na.beta_h * Na.h

Na.alpha_m := (25 – V) / (10 * (exp ((25 – V) / 10) – 1))

Na.beta_m := 4 * exp (- V / 18)

Na.alpha_h := 0.07 * exp (- V / 20)

Na.beta_h := 1 / (exp ((30 – V) / 10) + 1)

Na.G = 120

Na.E = 115

Hodgkin-Huxley Connection (to set up cable equations):

A = $connect (“Hodgin-Huxley Compartment”)

B = $connect (“Hodgin-Huxley Compartment”)

A.V' += (B.V – A.V) / R

B.V' += (A.V – B.V) / R

R = 10

The $connect() lines declare that “A” and “B” are instances of other parts. The exact names are arbitrary. The key characteristic of a connection is the ability to reference other instances and modify their variables.

Cable Model:

HH = $include (“Hodgkin Huxley”)

HH.$n = 3

C = $include (“Hodgkin-Huxley Coupling”)

C.A = HH

C.B = HH

C.$p = C.A.$index == C.B.$index - 1

A potential instance of connection C will be generated for every combination of two HH instances. The reserved variable $p determines whether that instance should actually exist. In this example, we are using $index to state that neighboring HH instances should link.

Here is the fully expanded equation set for Cable Model:

HH.$n = 3

HH.V’ = (HH.G * (HH.V_rest – HH.V) + HH.I_inj) / HH.C

HH.G = 0.3

HH.V_rest = 10.613

HH.C = 1

HH.K.$up.V’ += HH.K.I / HH.C

HH.K.I = HH.K.G * HH.K.n^4 * (HH.K.E – HH.V)

HH.K.n’ = HH.K.alpha_n * (1 – HH.K.n) – HH.K.beta_n * HH.K.n

HH.K.alpha_n := (10 – HH.V) / (100 * (exp ((10 – HH.V) / 10) - 1))

HH.K.beta_n := 0.125 * exp (-HH.V / 80)

HH.K.G = 36

HH.K.E = -12

HH.Na.$up.V’ += HH.Na.I / HH.C

HH.Na.I = HH.Na.G * HH.Na.m^3 * HH.Na.h * (HH.Na.E – HH.V)

HH.Na.m’ = HH.Na.alpha_m * (1 – HH.Na.m) – HH.Na.beta_m*HH.Na.m

HH.Na.h’ = HH.Na.alpha_h * (1 – HH.Na.h) – HH.Na.beta_h*HH.Na.h

HH.Na.alpha_m := (25 – HH.V) / (10 * (exp ((25 – HH.V) / 10) – 1))

HH.Na.beta_m := 4 * exp (- HH.V / 18)

HH.Na.alpha_h := 0.07 * exp (- HH.V / 20)

HH.Na.beta_h := 1 / (exp ((30 – HH.V) / 10) + 1)

HH.Na.G = 120

HH.Na.E = 115

C.A = HH

C.B = HH

C.A.V' += (B.V – A.V) / R

C.B.V' += (A.V – B.V) / R

C.R = 10

C.$p = C.A.$index == C.B.$index - 1

**Instantiation and Simulation**

This section describes a fairly specific procedure for simulation which is true mainly of the reference C backend. The purpose is to make the intended semantics clear, rather than to overly constrain how other backends behave. Simulation is on a “best effort” basis. A simulator should always try to run its closest approximation of the written model. It should provide warnings when the model contains elements that can’t be simulated according to these expectations. A simulator should only terminate with an error if the model is truly impossible to run, even in some limited form.

N2A reserves some variables to have special meaning during the instantiation of parts. All reserved variables start with the dollar-sign ($). These variables never resolve up the containment hierarchy, but instead have well-defined default values.

Before simulation, N2A fully expands the equation set for a model. It processes all $inherit() and $include() statements. For any $ equation that lacks an @ clause, it adds an @ $init. Thus a $ equation usually evaluates only once, during the first pass through the equation set when $init is set to 1. The user can change this by adding explicit conditions to it.

The equations of an instance are evaluated immediately when it is first created. Then a full time-step will go by before another evaluation. During the very first cycle of a simulation, parts are instantiated while $t is still 0. Thus, most instances will execute their init phase when $t is 0, and their first non-init cycle when $t advances by $dt. At init-time, almost all variables are 0 except for $index and $init. In particular, $dt is always 0 because no simulation time has passed yet.

The time-sequence for a simulation is:

$t = 0

instantiate new compartments based on $n

evaluate each equation set with $init=1 and $dt=0 (no sim-time has passed yet)

instantiate connections between new compartments

ditto

$t += $dt (amount of sim-time that has passed, not 0)

evaluate all current equation sets with $init=0

instantiate any new compartments and connections (same procedure as in $t=0)

$t += $dt …

The procedure for generating new instances in a population is:

For i = (previous value of $n) to (new value of $n-1)

Create a new instance of the part

Set all variables and implicit integrands to zero

Assign $index from pool of available numbers, else set $index = i

Set $init = 1

Evaluate $ equations

Evaluate non-$ equations

Set $init = 0

Most equation sets will assign a value to $xyz, giving the new instance a location in space. When $variables are evaluated at init time, only $index and $init are defined. If any initialization depends on the results of other initializations, then it is necessary to organize your equation set to use temporaries. Such temporary variables should not be prefixed with $.

At init time there is an exception to the instantaneous evaluation rule. $variables are evaluated first, then their values are made available to the non-$ equations. This allows non-$ variables to reference $variables, such as a calculated $xyz value.

The procedure for creating connections is:

Select the population to iterate over in the outer loop

Either random

Or its alias is specified by an assignment to $ref // must be a constant

For each newly created A in reference population

evaluate $xyz, the projection of A into the other population

if $xyz is undefined, then set to A.$xyz. If A.$xyz is undefined, then [0,0,0].

Note: $xyz must depend only on A. If $xyz is defined, then $ref must also be defined.

Filter subset of population B (criteria ANDed together):

Select $k nearest parts

Select parts within $radius of $xyz

Select all

Permute the subset, such that each A is paired with a different ordering over B

For all B in subset

If (connections of this type to A) >= A.$max, then stop iterating

If (connections of this type to B) >= B.$max, then skip to next B

Evaluate equation for $p with “$init” in @ clause

if $p > random draw with uniform distribution in [0,1)

Create new instance of connection

For each old A in “from” population

Do same as above, but only interact with newly created B in other population

note: If the expression for the connection's $xyz is invertible

then select a subset of old A for each new B.

Parts of the above procedure repeat as necessary until every A has connections >= A.$min and every B has connections >= B.$min. However, it is possible to specify a network where all members of one population reach their $max before all members of the other reach their $min. In this case, the simulation will continue with $min unfulfilled.

Sometimes a population connects to itself. This raises the question of whether an individual instance can connect to itself, or only to its neighbors. To test whether both ends of a connection are the same instance, compare their aliases. For example:

$p = A != B // only true if A and B refer to different instances

**Operators**

| Associativity | Precedence | Operator | Description |
| --- | --- | --- | --- |
| Left to Right | 1 | . | Namespace delimiter |
|  |  | *func*() | Function call |
|  |  | [] | Subscripts  Matrix constants |
|  |  | () | Override precedence |
|  |  | ' | Matrix transpose  Abbreviation for □\|$t (derivative w.r.t. time) |
|  |  | \| | Delimit two state variables in an ODE or PDE |
| Right to Left | 2 | - | Unary minus (makes a negative number) |
|  |  | ! | Logical NOT |
| Left to Right | 3 | ^ | Exponentiation |
|  | 4 | * | Multiply |
|  |  | / | Divide |
|  |  | % | Modulo |
|  | 5 | + | Add |
|  |  | - | Subtract |
|  | 6 | < | Less than |
|  |  | <= | Less than or equal to |
|  |  | > | Greater than |
|  |  | >= | Greater than or equal to |
|  | 7 | == | Equal to |
|  |  | != | Not equal to |
|  | 8 | && | Logical AND |
|  | 9 | \|\| | Logical OR |
|  | 10 | , | Separate expressions in a list, or elements in a row of a matrix. |
|  | 11 | ; | Separate rows in a matrix |
| None | 12 | = | Define a state variable |
|  |  | := | Define a temporary variable |
|  | 13 | @ | Separate assignment expression from conditional expression. |

**Types**

**float** – Floating point value. The precision is determined by the backend, and in some case may be specified by the user. All computation is done in this format. In particular, integer and Boolean values are treated as floats. Boolean true is 1 and false is 0.

**matrix** – A 2D raster of floats. Used primarily for specifying and manipulating coordinates. A matrix constant has the following format: [row1; row2; ... ; rowN]'. The trailing apostrophe (') is optional, and indicates that the matrix should be transposed. Rows are separated by semicolons. Values within a single row are separated by spaces. Any values in a row that are not specified are treated as zero. Two semicolons with nothing but white-space between them are treated as one semicolon. The number of columns in the matrix is determined by the longest row. Thus, an entire row of zeroes may be specified with a single 0 between semicolons.

**instance** – The data of a part or connection as it exists at runtime. A part can have any number of instances. An “alias” in a connection is a variable of type “instance”.

**Special Variables**

| $dt | Read: seconds since last evaluation of this instance.  Write: the preferred evaluation period for this instance. On simulators that support it, this may move the instance to a different position in the event queue, so its next update may come at a completely different time than its peers. |
| --- | --- |
| $index | An integer indicating the position of this instance in the population organized as a 1D array. Not defined for connections. |
| $init | Evaluates to 1 when instance is being constructed. Evaluates to 0 at all other times. |
| $k | An integer number of nearest neighbors to search for potential connections. If undefined or 0, then treated as infinite. |
| $live | Evaluates to 1 if this instance (generally a connection) currently exists, and to 0 if this instance is being evaluated for potential creation. |
| $max | Always prefixed by an alias in a connection equation set. Indicates the greatest number of instances from the other population that may connect to one instance of the aliased population. If undefined or 0, then treated as infinite. |
| $min | Always prefixed by an alias in a connection equation set. At least this many instances from the other population must connect to one instance of the aliased population. Default is 0. |
| $n | Quantity of instances to generate for the given population. Default is 1. |
| $p | Probability that a part exists. Decision is based on whether $p is greater than a random draw from a uniform distribution in [0, 1). A value of 0 forces a part to die, and a value of 1 forces it to live. If not specified, then the part always exists.  Connections: used to filter all possible combinations of two populations to determine those that will actually connect.  Compartments: used to implement population dynamics by randomly killing off instances. |
| $radius | A distance to search in the space of the “other” (non-reference) population for potential connections. If undefined or 0, then treated as infinite. Default is 0. |
| $ref | Which population to iterate over first when evaluating connections. If a connection references more than two populations, then they should be listed in order, separated by commas. |
| $t | Seconds of simulated time since start. |
| $type=A,B,… | Enables an instance to change into a different kind of part, or to split into instances of several different parts, analogous to cell-division and specialization. The right-hand-side of the assignment is a comma-separated list of part names. One or more of the destination parts are allowed to be the same as the current part.  Write: Create a new instance for each part listed on the right-hand-side. Each new instance first does a standard initialization, then all values with exactly the same name are copied over from the old instance. Values without matching names are lost. If at least one of the named parts is the same as the current part, the first one is considered to be fulfilled by the current instance. Otherwise it dies at the end of the cycle. A part that lives on retains its current connections, while all new parts must form new connections.  Read: If this instance was created by any process other than an assignment to $type, then the value reads 0. If this instance was born from a $type assignment, then the value is the one-based position in the list of parts on the right-hand-side. After one update cycle this value resets to 0. |
| $xyz | Spatial location of instance. For connections, the projection of an instance of the reference population into the space of the other population. |

**Pseudo-Functions**

| prefix = $connect(name,…) | Declares that “prefix” is an instance of a connected part. The list of part names is a hint about what this connection can handle. All descendants of a part are also implicitly allowed. A connection may be used against any part that has the right variable names, even it is not listed here. That is, N2A is not strictly typed. |
| --- | --- |
| prefix = $include(name) | Appends named equation set, while boxing them in the prefix. |
| parent = $inherit(name) | Appends named equation set directly. Variable on LHS is ignored, but must be unique within the containing equation set. |

**Predefined Functions**

All backends are expected to support these functions, though in practice some may not. If a backend does support a function listed here, then it must follow the given semantics exactly. Any function not listed here has arbitrary semantics defined by the backend, and is less likely to be portable.

| gauss(dimension) | Returns a random draw from a Gaussian distribution with mean 0 and variance 1.  dimension – Number of elements in resulting vector. If 1, then returns a scalar. Default is 1. |
| --- | --- |
| grid(i,sx,sy,sz,dx,dy,dz) | Returns a 3-vector indicating a point in space.  i -- the index of the current element.  sx,sy,sz -- the integer stride along each dimension. Unused dimensions should be set to 1.  dx,dy,dz -- the spatial distance between adjacent elements along each dimension. Unused dimensions should be set to 0.  Note that a stride is not the same thing as the number of elements along that dimension. It is instead the integer difference in the index between two elements along that dimension. For example, suppose the layout is a 4x4x4 cube, and that y is the major dimension (that is, all indices associated with a particular y value are contiguous), followed by z and finally x. The strides would be: sx=1, sy=16, sz=4 |
| pulse(x,width,period,rise,fall) | Generate square and triangular waves. Returns values in [0,1]. Exact shape of wave depends on input parameters.  x – Independent variable. In practice this is time or some function thereof. First rise starts at 0. Any negative value results in an output of 0.  width – Time from end of rise to start of fall. That is, how long the output remains at 1.  period – Amount of time between start of rise in one cycle and start of rise in next cycle. If 0, does not repeat. Default is 0.  rise – How long for output to change from 0 to 1. Default is 0 (instantaneous).  fall – How long for output to change from 1 to 0. Default is 0 (instantaneous). |
| trace(expression,“column name”) | Sends the value of expression to a table on standard-out. If “column name” is omitted, the associated column in the table gets an automatically-generated unique name based on part prefixes and indices. The result of this function is the value of the expression itself, so this may be used to wrap any part of a larger expression. |
| uniform(dimension) | Returns a random number in [0,1] with uniform distribution.  dimension – Number of elements in resulting vector. If 1, then returns a scalar. Default is 1. |
|  |  |
